# Phosphatidylethanolamine and phosphatidylcholine biosynthesis by the Kennedy pathway occurs at different sites in *Trypanosoma brucei*

**DOI:** 10.1038/srep16787

**Published:** 2015-11-18

**Authors:** Luce Farine, Moritz Niemann, André Schneider, Peter Bütikofer

**Affiliations:** 1 Institute of Biochemistry and Molecular Medicine, University of Bern, 3012 Bern, Switzerland; 2 Graduate School for Cellular and Biomedical Sciences, University of Bern, 3012 Bern, Switzerland; 3 Department of Chemistry and Biochemistry, University of Bern, 3012 Bern, Switzerland

## Abstract

Phosphatidylethanolamine (PE) and phosphatidylcholine (PC) are among the most abundant phospholipids in biological membranes. In many eukaryotes, the CDP-ethanolamine and CDP-choline branches of the Kennedy pathway represent major and often essential routes for the production of PE and PC, with ethanolamine and choline/ethanolamine phosphotransferases (EPT and CEPT, respectively) catalysing the last reactions in the respective pathways. Although the site of PE and PC synthesis is commonly known to be the endoplasmic reticulum (ER), detailed information on the localization of the different phosphotransferases is lacking. In the unicellular parasite, *Trypanosoma brucei*, both branches of the Kennedy pathway are essential for cell growth in culture. We have previously reported that *T. brucei* EPT (TbEPT) catalyses the production of ether-type PE molecular species while *T. brucei* CEPT (TbCEPT) synthesizes diacyl-type PE and PC molecular species. We now show that the two enzymes localize to different sub-compartments of the ER. By expressing a series of tagged forms of the two enzymes in *T. brucei* parasites, in combination with sub-cellular fractionation and enzyme activity measurements, TbEPT was found exclusively in the perinuclear ER, a distinct area located close to but distinct from the nuclear membrane. In contrast, TbCEPT was detected in the bulk ER.

Phosphatidylcholine (PC) and phosphatidylethanolamine (PE) are the most abundant glycerophospholipids in eukaryotes^1^,^2^. Besides their roles as main structural components of all cellular membranes, PC and PE fulfil various other functions. PC is an important source for the formation of second messengers and lipid mediators^3^,^4^. Disturbance of its production interferes with cell proliferation, differentiation and membrane movement throughout the cell^1^. On the other hand, PE plays essential roles in autophagy, cell division and protein folding, and represents a precursor for the synthesis of several protein modifications^5^,^6^. In addition, both PC and PE are intermediates in the synthesis of other glycerophospholipid classes^7^,^8^.

*De novo* formation of PE and PC in eukaryotes occurs via several pathways. PE can be synthesized by the CDP-ethanolamine branch of the Kennedy pathway^9^, decarboxylation of phosphatidylserine (PS)^5^ and base exchange with PS^10^, while PC is formed via the CDP-choline branch of the Kennedy pathway^9^ and methylation of PE^11^. In addition to *de novo* synthesis, PE and PC can be generated by acylation of *lyso*-phospholipids taken up by cells from the environment^12^,^13^.

The Kennedy pathway plays a central role in both PE and PC synthesis. Although it is not essential for growth of yeast under normal conditions^14^, the pathway is indispensable for mammalian development^15^ and survival of various unicellular eukaryotes^16^. In the CDP-ethanolamine branch, ethanolamine kinase catalyses the ATP-dependent phosphorylation of ethanolamine to ethanolamine-phosphate, which in turn becomes activated to CDP-ethanolamine by ethanolamine-phosphate cytidylyltransferase. In analogy, in the CDP-choline branch, choline is phosphorylated and activated to CDP-choline by choline kinase and choline-phosphate cytidylyltransferase, respectively. The final reactions in PE and PC formation by the Kennedy pathway are catalysed by enzymes with variable substrate specificities: i) CPT enzymes are highly specific for the CDP-choline branch, leading to formation of PC, ii) CEPT enzymes show a dual specificity for CDP-choline and CDP-ethanolamine, resulting in the formation of PC and PE, respectively, and iii) EPT enzymes exhibit high specificity for CDP-ethanolamine, leading to PE formation. Although CPT enzymes (hCPT1^17^ and ScCPT1^18^) and CEPT enzymes (hCEPT1^17^, AtAAPT1 and 2^19^ and TbCEPT^20^) have been identified and characterized in some cells, their specificities are poorly defined. hEPT1^21^ and TbEPT^20^ seem to be specific for PE formation, while ScEPT1^18^ and CrEPT^22^ are also able to synthesize PC to a lesser extent.

The localization of CPT, CEPT and EPT enzymes defines the sites of PC and PE production in a cell. However, only few reports have addressed this crucial question and, thus, the enzymes’ sub-cellular distribution has largely remained elusive. In mammalian cells, CPT1 has been localized to the Golgi, while CEPT1 was found in the endoplasmic reticulum (ER) and nuclear envelope^23^. In contrast, yeast CPT1 was enriched in microsomal fractions and in the Golgi^24^,^25^, whereas a GFP-tagged version of CPT1 didn’t co-localize with any known organelle marker^26^. In addition, GFP-tagged yeast EPT1 revealed a punctate staining, showing at least partial co-localization with a Golgi marker^26^. More recently, immunofluorescence studies in *Toxoplasma gondii* showed co-staining of two putative EPT enzymes with an ER marker^27^. However, the substrate specificities and products of the two enzymes have not been determined, i.e. it is unclear if they represent CEPT or EPT enzymes.

*Trypanosoma brucei* is the causative agent for Human African Trypanosomiasis (HAT) and *nagana*, the equivalent animal disease. These diseases are lethal if untreated and represent a major cause of poverty in sub-Saharan Africa^28^. Unfortunately, currently available drugs can cause serious secondary effects and lack efficacy against certain forms of the disease, thus the identification of novel drug targets and development of new compounds to combat trypanosomes represent a major focus of the research community^29^,^30^,^31^. Two replicative stages of *T. brucei* are studied most frequently, the procyclic form of the insect midgut and the long slender bloodstream form of the mammalian host. Both parasite life stages can be grown easily in culture. *T. brucei* has also been used as valuable model eukaryote to study various biological processes, including phospholipid biosynthesis^6^,^32^. The lipid composition of *T. brucei* is similar to that of other eukaryotes, with PC (45–60%) and PE (10–20%) representing the major glycerophospholipid classes^33^,^34^. Interestingly, and in contrast to many other eukaryotes, *T. brucei* glycerophospholipids contain very high amounts of ether-type phospholipid subclasses^35^,^36^, in particular PE, which consists of >80% and >60% of ether-type molecular species in procyclic and bloodstream forms, respectively^20^,^35^,^36^. Although *T. brucei* parasites can acquire lipids from the environment, *de novo* phospholipid synthesis is essential for parasite survival of both bloodstream and procyclic forms^33^,^34^. The Kennedy pathway for PE and PC synthesis has been studied in detail in *T. brucei*. The enzymes of the CDP-ethanolamine and CDP-choline branches are expressed in both bloodstream and procyclic forms and have been studied experimentally^20^,^37^,^38^. Although other biosynthetic routes for PE synthesis are active in *T. brucei*, trypanosome growth depends on a functional Kennedy pathway for both PE and PC formation, making the individual enzymes of the pathway interesting potential drug targets to inhibit parasite proliferation^16^.

Our previous study in *T. brucei* procyclic forms using RNA interference to separately knock down *EPT* and *CEPT* was the first to show in a eukaryote that TbEPT is responsible for the synthesis of ether-type PE molecular species whereas TbCEPT mediates the formation of diacyl-type PE and all PC molecular species (Fig. 1)^20^. Interestingly, while the two enzymes use the same hydrophilic substrate, i.e. CDP-ethanolamine, for PE production, the hydrophobic substrates are different. Synthesis of ether-type molecular species by EPT requires alk-1-enyl-acylglycerol or alkyl-acylglycerol (both referred to as AAG in the present study), while diacyl-type molecular species formation by CEPT requires diacylglycerol (DAG) as substrate. In general, the synthesis of the different hydrophobic substrates is thought to occur in different compartments in eukaryotes. While the formation of AAG seems restricted to peroxisomes^39^, or similar specialized compartments in other cells such as glycosomes in *T. brucei*^40^,^41^, DAG can be produced at several locations. The goal of the present work was, using *T. brucei* as eukaryotic model organism, to study the sub-cellular localization of EPT and CEPT, which catalyse the last step of the two branches of the Kennedy pathway, in a single cell. By expressing several differently tagged forms of TbEPT and TbCEPT and their subsequent analysis using immunofluorescence, sub-cellular fractionation and enzyme activity measurements, we found that TbEPT localizes exclusively to perinuclear membranes while TbCEPT shows a dual distribution between the ER and the perinuclear region. These findings have important implications on the availability of the substrates for PE and PC synthesis, AAG and DAG, in the respective compartments.

## Results and Discussion

According to their *in vitro* and *in vivo* substrate specificities, EPT/CEPT and CEPT/CPT enzymes catalyse the last step in the two branches of the Kennedy pathway, leading to the formation of the most abundant eukaryotic glycerophospholipid classes PE and PC, respectively. Although scattered data about the sub-cellular localization of CPT, CEPT and EPT in mammalian cells, yeast and *Toxoplasma* is available^23^,^24^,^25^,^26^,^27^, the results are conflicting and provide no clear information on the site(s) of *de novo* synthesis of PC and PE. In particular, a detailed study to localize these enzymes in a single cell is lacking. In *T. brucei* parasites, PE and PC synthesis by the Kennedy pathway is essential and the substrate specificities of TbEPT and TbCEPT have been characterized using RNAi cell lines to down-regulate the enzymes individually^20^,^38^. In the present study, we have analysed the localization of TbCEPT and TbEPT by immunofluorescence microscopy of differently tagged forms of the enzymes, and by sub-cellular fractionation and enzyme activity measurements.

### Localization of tagged forms of TbEPT and TbCEPT

**Figure 2:**
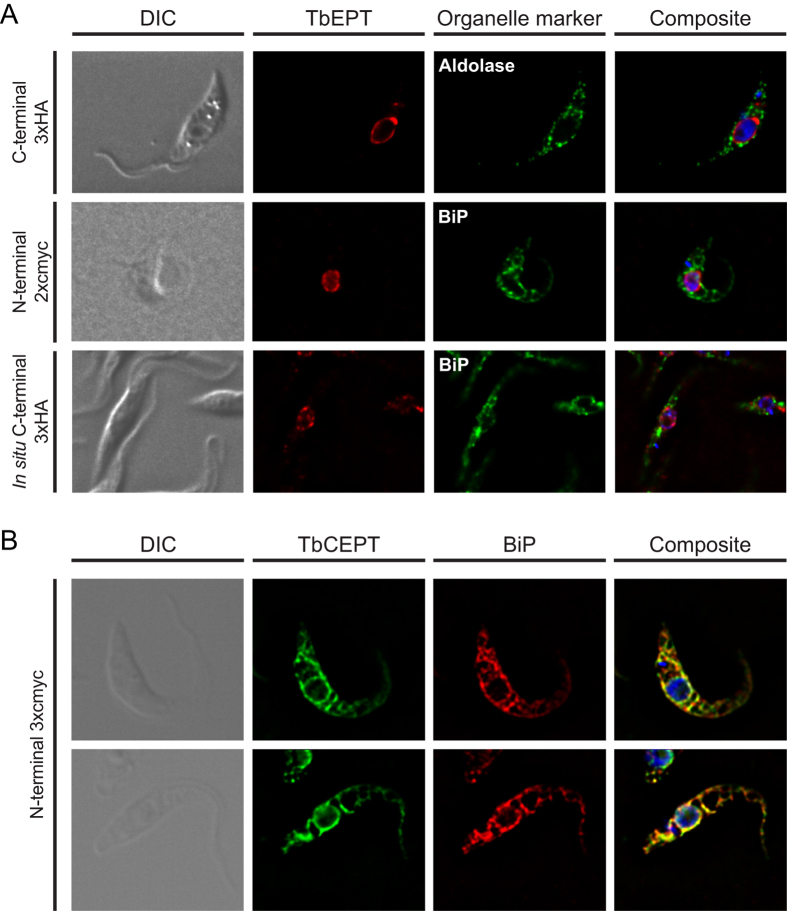
Sub-cellular localization of TbEPT and TbCEPT by immunofluorescence microscopy. *T. brucei* procyclic forms expressing tagged versions of (**A**) TbEPT or (**B**) TbCEPT (with the types and positions of the tags indicated in the left margin) were immunostained with antibodies against HA or cmyc, together with antibodies against aldolase (glycosomal marker) or BiP (luminal ER marker), in combination with the respective fluorescence-conjugated secondary goat anti-mouse and anti-rabbit antibodies. DNA in the composite pictures was stained with DAPI. DIC, differential interference contrast.

Based on its previously reported specificity for the synthesis of ether-type PE molecular species in *T. brucei* procyclic forms^20^, we expected TbEPT to localize in glycosomes, which are the site of ether-type (alk-1-enyl-acylglycerol and alkyl-acylglycerol) precursor synthesis^40^. In contrast, we found that expression of a C-terminally 3xHA-tagged version of TbEPT (TbEPT-3xHA) showed a perinuclear staining, but no co-localization with the glycosomal marker aldolase (Fig. 2A, top panels). A similar staining was also observed in parasites expressing an N-terminally 2xcmyc-tagged TbEPT or an *in situ* C-terminally 3xHA-tagged form of the enzyme (Fig. 2A, middle and bottom panels, respectively). Remarkably, we observed little or no co-localization of TbEPT with the ER marker BiP. The exclusive perinuclear localization of TbEPT was independent of the position of the tag (N- or C-terminal), the nature of the tag (HA, cmyc or GFP (see below)) or the method used to express the tagged protein (inducible *versus* stable expression), and was seen in >95% of cells, indicating that the results likely reflect the genuine localization of TbEPT. Together, these results indicate that TbEPT is not localized in the bulk ER, i.e. the site of PE formation in other eukaryotes, but in a sub-compartment in close proximity to the nuclear membrane.

In addition, we analysed the sub-cellular localization of TbCEPT in *T. brucei* procyclic forms by expressing an N-terminally 3xcmyc-tagged version of the enzyme. The results show an essentially complete co-localization of TbCEPT with the ER marker BiP (Fig. 2B).

**Figure 3:**
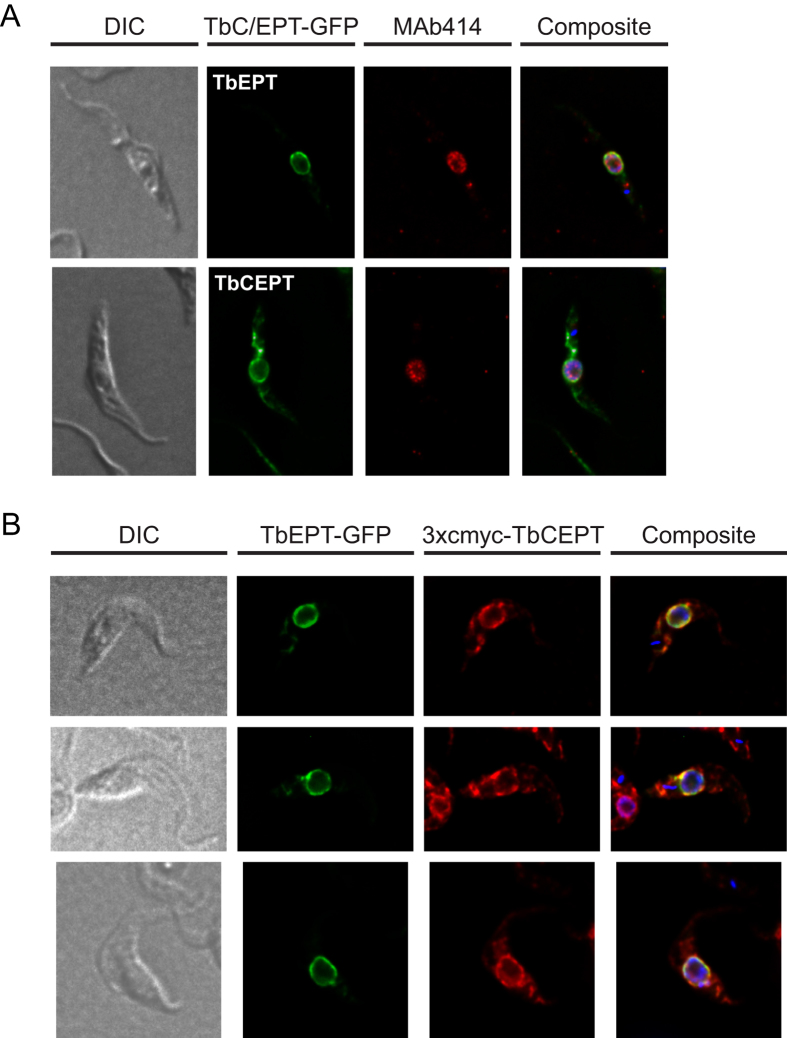
Localization of tagged forms of TbEPT and TbCEPT in the same cell. Panel (**A**) GFP-tagged TbEPT (top panels) and TbCEPT (bottom panels) were transiently expressed in *T. brucei* procyclic forms and co-stained with MAb414, a nuclear pore complex marker. Panel (**B**) GFP-tagged TbEPT was transiently expressed in procyclic forms stably expressing 3xcmyc-TbCEPT and cells were immunostained with anti-cmyc antibody and fluorescence-conjugated goat anti-mouse antibody. DNA in the composite pictures was stained with DAPI. DIC, differential interference contrast.

Because of the close apposition of the nuclear membrane and the region of the ER closest to the nuclear membrane, the unique perinuclear staining pattern of TbEPT was further investigated. To distinguish between nuclear and ER membranes, procyclic forms expressing TbEPT and TbCEPT whose C-termini were fused to GFP were co-stained with an antibody (MAb414) directed against nuclear pore complex proteins. TbEPT-GFP showed the above mentioned ring-like distribution around the nucleus (Fig. 3A; compare with Fig. 2A). However, although staining with MAb414 revealed a similar ring-like staining pattern, little co-localization with TbEPT-GFP was observed (Fig. 3A). In addition, in agreement with the staining of 3xcmyc-TbCEPT (Fig. 2B), TbCEPT-GFP showed staining of the bulk ER. Again, as for TbEPT-GFP, little co-localization was observed between TbCEPT-GFP and MAb414.

### Localization of TbEPT and TbCEPT in a single cell

To localize both TbEPT and TbCEPT in a single parasite, TbEPT-GFP and 3xcmyc-TbCEPT were co-expressed and analysed by (immuno)fluorescence microscopy (Fig. 3B). The results show that the two enzymes co-localize in the ER region proximal to the nucleus, however, while TbEPT is confined to the perinuclear region only, TbCEPT is distributed throughout the ER network. Together, our results demonstrate that TbEPT is located exclusively in an ER sub-domain in close proximity but distinct from the nuclear membrane. In contrast, TbCEPT reveals a staining pattern typical for bulk ER, although a sub-fraction co-localizes with TbEPT.

### Sub-cellular fractionation and enzyme activity measurements

**Figure 4:**
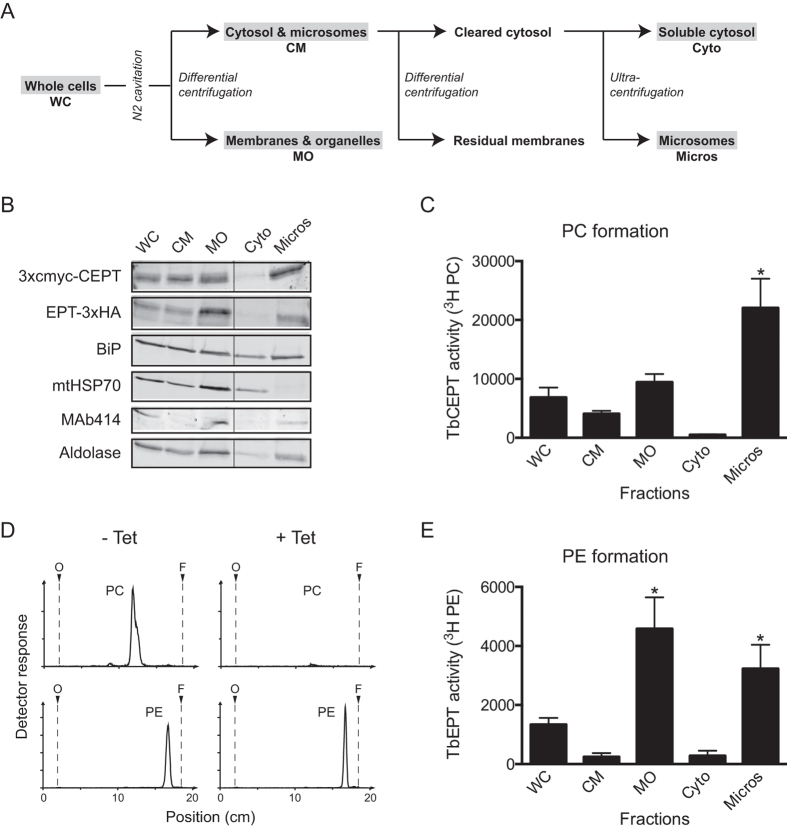
Sub-cellular distribution of TbCEPT and TbEPT protein and enzyme activity. Panel (**A**) Preparation of sub-cellular fractions. Procyclic trypanosomes were broken up by N_2_ cavitation and fractionated by differential centrifugation as indicated. Whole cell lysate (WC), cytosol and microsomes (CM), membranes and organelles (MO), soluble cytosol (Cyto) and microsomal (Micros) fractions were subsequently analysed for the presence of tagged TbCEPT and TbEPT and enzyme activity. Panel (**B**) SDS-PAGE and Western blot analysis of sub-cellular fractions from a mixed culture of trypanosomes expressing 3xcmyc-CEPT or EPT-3xHA. Proteins were detected using antibodies against HA, cmyc, BiP (as ER marker), mtHSP70 (as mitochondrial marker), MAb414 (as nuclear envelope marker) and aldolase (as glycosomal marker). Panel (**C**) TbCEPT activity in sub-cellular fractions as determined using [^14^C]CDP-choline as substrate and TLC to separate the products. All fractions contained identical amounts of protein. [^14^C]PC formation was quantified by scanning the TLC plates using a radioisotope detector. The values represent means ± standard deviations from 3 experiments. The asterisk indicates a significant difference in TbCEPT activity between the microsomal fraction (Micros) and all other fractions (p < 0.05, Student’s t-test). Panel (**D**) [^3^H]PC and [^3^H]PE formation in TbCEPT RNAi trypanosomes. Control (-Tet) and tetracycline-induced (+Tet) cells, resulting in depletion of TbCEPT, were incubated in presence of [^3^H]choline or [^3^H]ethanolamine and formation of [^3^H]PC and [^3^H]PE, respectively, was detected after lipid extraction and TLC analysis using a radioisotope detector. Panel (**E**) TbEPT activity in sub-cellular fractions of TbCEPT-depleted trypanosomes as determined using [^14^C]CDP-ethanolamine as substrate and TLC to separate the products. All fractions contained identical amounts of protein. [^14^C]PE formation was quantified by scanning the TLC plates using a radioisotope detector. The values represent means ± standard deviations from 3 experiments. The asterisks indicate a significant difference in TbEPT activity between the microsomal (MO) fraction, or the membranes and organelles (Micros) fraction, and the WC, CM and Cyto fractions (p < 0.05, Student’s t-test). WC, whole cells; CM, cytosol and microsomes; MO, membranes and organelles; Cyto, soluble cytosol; Micros, microsomes.

The sub-cellular localization of TbEPT and TbCEPT was also investigated using cell fractionation of different trypanosome cell lines. To compare the sub-cellular distribution of the tagged enzymes with endogenous TbCEPT activity, a mixed culture of *T. brucei* procyclic forms expressing either TbEPT-3xHA or 3xcmyc-TbCEPT was fractionated by differential centrifugation (Fig. 4A). Subsequently, in each sub-cellular fraction the presence of the tagged enzymes was analysed by SDS-PAGE and immunoblotting (Fig. 4B) and the enzymatic activity of TbCEPT was determined (Fig. 4C). The results show the highest enrichment of 3xcmyc-TbCEPT in the microsomal fraction, while TbEPT-3xHA was enriched the most in the membranes and organelles fraction (Fig. 4B). These findings support the immunofluorescence results showing that the two tagged enzymes localize differently in *T. brucei*. The distribution of marker proteins for mitochondria (mtHSP70), ER (BiP) and glycosomes (aldolase) in the different fractions confirms previous results^42^ and shows that the microsomal fraction is depleted of mitochondrial and nuclear (MAb414) membranes, while it contains the markers for ER and glycosomes (Fig. 4B). Determination of TbCEPT activity using [^14^C]CDP-choline as substrate in the fractions after differential centrifugation showed highest [^14^C]PC formation in the microsomal fraction (Fig. 4C). This result is in line with the enrichment of 3xcmyc-TbCEPT in the same fraction (Fig. 4B). Since TbCEPT activity was identical in parasites before and after induction of 3xcmyc-TbCEPT expression (results not shown), indicating that the tagged enzyme doesn’t contribute significantly to endogenous TbCEPT activity or that 3xcmyc-TbCEPT while correctly localized is not active, the measured *in vitro* activity represents endogenous TbCEPT activity.

To determine the sub-cellular distribution of endogenous TbEPT activity, an RNAi cell line allowing down-regulation of TbCEPT expression^20^ was used for the following reason. TbCEPT shows dual substrate activity, i.e. it not only catalyses the production of PC but also PE^20^ and, thus, its activity contributes to [^14^C]PE formation when using [^14^C]CDP-ethanolamine as substrate. Therefore, to allow determination of TbEPT activity in the absence of TbCEPT, TbCEPT mRNA was silenced using tetracycline-inducible RNAi. Absence of TbCEPT activity was confirmed by the inability of TbCEPT-depleted parasites to form [^3^H]PC using [^3^H]choline as substrate (Fig. 4D, upper panels). In contrast, TbEPT-mediated formation of [^3^H]PE using [^3^H]ethanolamine as substrate was unchanged in TbCEPT-depleted parasites (Fig. 4D, lower panels). These results are in line with previous data^20^. Analysis of [^14^C]PE formation in the fractions after differential centrifugation from TbCEPT-depleted parasites revealed highest TbEPT activity in the membranes and organelles and microsomal fractions (Fig. 4E). The result is in good agreement with the enrichment of TbEPT-3xHA in these fractions (Fig. 4B).

**Figure 5:**
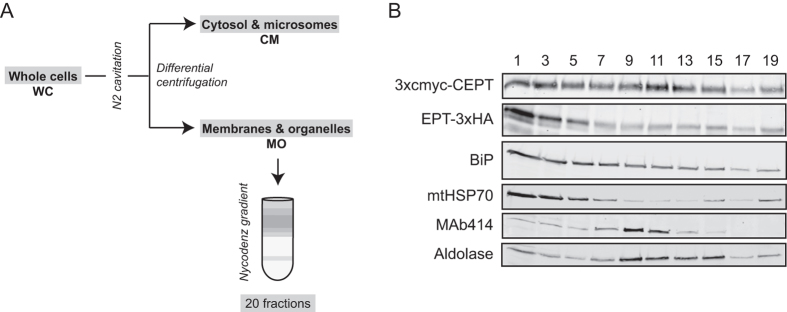
Fractionation of TbCEPT and TbEPT by gradient centrifugation. Panel (**A**) The membranes and organelles fraction, obtained after breaking up the cells followed by a centrifugation step, was further separated using a linear Nycodenz gradient. Fractions collected from the top of the gradient were then analysed for the presence of tagged TbCEPT and TbEPT. Panel (**B**) SDS-PAGE and Western blot analysis of odd-numbered fractions from a mixed culture of trypanosomes expressing 3xcmyc-CEPT or EPT-3xHA. Proteins were detected using antibodies against HA, cmyc, BiP (as ER marker), mtHSP70 (as mitochondrial marker), MAb414 (as nuclear envelope marker) and aldolase (as glycosomal marker).

Further fractionation of the membranes and organelles fraction, containing 3xcmyc-TbCEPT and TbEPT-3xHA, using a linear 18–50% Nycodenz gradient (Fig. 5A) again revealed a different distribution pattern for the two enzymes (Fig. 5B). TbEPT-3xHA co-fractionated most closely with BiP and mtHSP70 at the top of the gradient, while 3xcmyc-TbCEPT showed a dual enrichment in the upper (fractions 1–3) and middle fractions (fractions 11–13). While the distribution of 3xcmyc-CEPT partly overlapped with MAb14 and aldolase, the fractions showing the highest enrichment of the respective enzymes are clearly different. These results are consistent with the immunofluorescence pictures showing that TbEPT and TbCEPT are localized differently and are not enriched in glycosomal or nuclear membranes (Figs 2 and 3).

## Conclusions

In *T. brucei* parasites, TbEPT and TbCEPT catalyse the last step in the CDP-ethanolamine and CDP-choline branches of the Kennedy pathway and, thus, determine the site(s) of synthesis of the two major glycerophospholipid classes PE and PC, respectively. Both pathways are essential for growth of parasites in culture^20^,^38^. Our work using immunofluorescence microscopy and immunoblotting to localize differently tagged forms of TbEPT and TbCEPT, in combination with sub-cellular fractionation and activity measurements to determine the distribution of the endogenous enzymes, showed that both TbEPT and TbCEPT are present in the ER. However, TbEPT is confined to the perinuclear region of the compartment while TbCEPT is distributed throughout the entire ER network. The reactions catalysed by TbEPT and TbCEPT, both of which are predicted to have multiple transmembrane domains and thus are tightly membrane-bound, combine water-soluble CDP-activated precursors with hydrophobic substrates, i.e. DAG and/or AAG. Although the site(s) of DAG production in trypanosomes has not been studied, it is generally believed that DAG is available in all membranes. In contrast, production of AAG in *T. brucei* has been localized in glycosomes^40^. Our observation that TbEPT is localized exclusively in the perinuclear ER implies that the hydrophobic substrate of the reaction, AAG, is available in this sub-compartment, or in very close proximity. At present, it is not known if a sub-population of glycosomes associates with the perinuclear ER or if AAG can be trafficked from glycosomes to other organelles. Mechanisms to transfer AAG from glycosomes to the perinuclear ER may involve protein- or vesicle-mediated transport, or direct membrane contact sites.

EPT and CEPT/CPT enzymes are present in essentially all eukaryotic cells. It will be interesting to study if the two enzymes show a similar intracellular distribution in other unicellular organisms, or mammalian cells.

## Methods

Unless otherwise stated, all reagents were of analytical grade and purchased from Sigma-Aldrich (Buchs, Switzerland) or Merck (Darmstadt, Germany). Restriction enzymes were obtained from Fermentas (St. Leon-Rot, Germany) and antibiotics from Sigma-Aldrich, Invivogen (Nunningen, Switzerland) or Invitrogen (Basel, Switzerland). [^14^C]CDP-choline (methyl-[^14^C]) and [^14^C]CDP-ethanolamine (ethanolamine-1,2-[^14^C]) (both 0.1 mCi/ml, 55 mCi/mmol), and [^3^H]choline and [^3^H]ethanolamine (both 1 mCi/ml, 60 mCi/mmol) were purchased from American Radiolabeled Chemicals (St. Louis, USA).

### Trypanosomes and culture conditions

*T. brucei* procyclic form strain 427 was cultured at 27 °C in SDM-79^43^ containing 5% heat-inactivated fetal bovine serum (Invitrogen). *T. brucei* procyclic form strain 29–13 co-expressing a T7 polymerase and a tetracycline repressor^44^ (obtained from Paul Englund, Baltimore, MD) was cultured at 27 °C in SDM-79 containing 10% heat-inactivated fetal bovine serum, 25 μg/mL hygromycin, and 15 μg/mL G418.

### Generation of tagged TbCEPT and TbEPT

To generate C-terminally GFP (green fluorescent protein)-tagged forms of *TbEPT* (Tb927.10.13290) and *TbCEPT* (Tb927.10.8900), the corresponding open reading frames were amplified by PCR using primers EPT_GFP_Fw GCCCAAGCTTATGGGAGTAGTAGACTTACTCACTTCT and EPT_GFP_Rv GCCGCTCGAGTCCACTTTCCTTACGGAGCCCT, and CEPT_GFP_Fw GCCCAAGCTTATGAACGCGCCTCACAGAACTC and CEPT_GFP_Rv GCCGCTCGAGTCCCACTTCGTGTTGGAGTCATG (restriction sites underlined), respectively. The products were ligated into HindIII- and XhoI-digested plasmid pG-EGFPΔLII b^45^, resulting in plasmids pLFeptGFP and pLFceptGFP. To generate C-terminally 3xHA (hemagglutinin)-tagged *TbEPT* and N-terminally 3xcmyc-tagged *TbCEPT*, the corresponding open reading frames were amplified by PCR using primers EPT_GFP_Fw and EPT_HA_Rv CGCTCTAGACTCCACTTTCCTTACGGAGCC, and CEPT_cmyc_Fw GCCCAAGCTTAACGCGCCTCACAGAGCTC and CEPT_cmyc_Rv GCGGGATCCCTACTCCCACTTCGTGTTGGCG, respectively. The products were ligated into two vectors: i) HindIII- and XhoI-digested plasmid pAG3020-3^46^, resulting in plasmid pLFeptHA, and ii) HindIII- and BamHI-digested plasmid pJM2^47^, resulting in plasmid pLFceptCMYC, respectively. Generation of N-terminally 2xcmyc tagged *TbEPT* was done by amplification of *TbEPT* open reading frame using primer EPT_cmyc_Fw containing 2xcmyc CCCGAAGCTTATG*GAGCAGAAGCTCATTTCTGAGGAGGACCTTGAGCAGAAGCTCATTTCTGAGGAGGACCTT*CTCGAGGGAGTAGTAGACTTACTCACTTCTACGCG (2xcmyc sequence in italic) and primer EPT_cmyc_Rv CGCGGATCCTCACTCCACTTTCCTTACGGAGCCCT. The product was ligated into HindIII- and BamHI-digested plasmid pALC14 (derivative of pLew100^44^) resulting in plasmid pLFeptCMYC. To generate C-terminal *in situ* tagged *TbEPT*, a one-step PCR strategy with the vector pMOTag3H was followed as described previously^48^.

### Stable and transient transfection of trypanosomes

Trypanosomes were harvested at mid-log phase, washed once in phosphate-buffered saline (PBS; 137 mM sodium chloride, 2.7 mM potassium chloride, 10 mM disodium phosphate, 2 mM monopotassium phosphate, pH 7.4) and resuspended in 100 μl TbBSF buffer (90 mM sodium phosphate, 5 mM potassium chloride, 0.15 mM calcium chloride, 50 mM HEPES, pH 7.3)^49^ previously mixed with 10 μg of plasmid or 4 μg of purified PCR product (*in situ* tag). Electroporation was performed in 100 μl nucleocuvettes using Lonza 4D Nucleofector System (pulse code FI-115, “Primary Cell P3” solution). GFP constructs were expressed transiently (circular plasmids were transfected into 427 strain and slides were prepared 18–24 hours after transfection) whereas HA and cmyc constructs were stably transfected into 29–13 strain (plasmids were linearized with NotI prior to transfection). Clones were obtained by limited dilution and selected in the presence of the corresponding antibiotic, 2 μg/ml puromycin for HA and cmyc constructs, or 15 μg/ml G418 for the *in situ* construct. Proper integration of the constructs was confirmed by PCR using primers binding upstream of the recombination site and downstream of the inserted gene. Expression of HA- or cmyc-tagged proteins was induced by addition of 1 μg/ml tetracycline to the culture medium.

### Fluorescence microscopy

Trypanosomes were collected and washed once in cold PBS before being allowed to adhere onto microscopy slides (Thermo Scientific) for 10 min. Cells were fixed with 4% paraformaldehyde, washed once with cold PBS and permeabilized with 0.2% (w/v) Triton X-100 in PBS. Blocking was performed with 2% bovine serum albumin in PBS (blocking solution) for 30 min, followed by 45 min incubation of first antibody diluted in blocking solution. The following antibodies were used: mouse monoclonal α-cmyc 9E10 (Santa Cruz Biotechnology; diluted 1:200), mouse monoclonal α-HA.11 16B12 (Covance, diluted 1:250), rabbit α-BiP (a kind gift of James D. Bangs, University of Buffalo, Buffalo, NY; diluted 1:2500), rabbit α-aldolase (a kind gift of Paul Michels, University of Edinburgh, Edinburgh, Scotland; diluted 1:5000), and mouse α-nuclear pore complex proteins (MAb414, Covance; diluted 1:5000). After washing, the corresponding secondary antibodies goat anti-mouse and anti-rabbit AlexaFluor 594 and 488 (Invitrogen; diluted 1:800) in blocking solution were added for 45 min. For the preparation of GFP-expressing cells, slides were kept protected from light during the whole procedure. After washing, cells were mounted with Vectashield containing 4′,6-diamidino-2-phenylindole (DAPI, Vector Laboratories). Fluorescence microscopy was performed with a Leica DMI6000 B inverted microscope and pictures were acquired, processed and 3D-deconvolved with LAS X software (Leica Microsystems CMS GmbH).

### Cell fractionation

Following a previously published procedure^50^, parasites were fractionated into a crude microsomes and a membranes and organelles fraction, the latter of which was subsequently fractionated using a Nycodenz gradient. Briefly, 2–4 × 10^10^ cells were harvested by centrifugation and subjected to nitrogen cavitation at isotonic conditions. Membranes and organelles (containing most organelles) were separated from cytosol and microsomes (crude microsomes) by differential sedimentation. The membranes and organelles fraction was subjected to DNase treatment, resuspended in 80% Nycodenz, loaded underneath a 18–50% (w/v) linear Nycodenz gradient using a syringe and centrifuged for 45 min in a SW28 rotor at 27000 rpm at 4 °C. Fractions (20 × 2 ml) were collected from the top of the gradient. The crude microsomal fraction was diluted 1:4 in HKM buffer (50 mM HEPES, 25 mM KCl; 5 mM MgCl_2_·6 H_2_O) and a clearing spin was carried out (30 min at 4 °C at 30000 × *g* in a SS34 rotor) to eliminate residual mitochondrial vesicles. Microsomes were collected from this cleared supernatant by ultracentrifugation (2 h at 4 °C at 100000 × *g* in a SW28 rotor). The microsomal fraction has previously been called crude ER fraction^42^.

### SDS-polyacrylamide gel electrophoresis (SDS-PAGE) and immunoblotting

Protein concentrations were determined using a BCA Protein Assay Kit (Pierce). Protein (10 μg) from each fraction was diluted in SDS sample buffer, separated by SDS-PAGE using 12% polyacrylamide gels under reducing conditions^51^, and transferred onto nitrocellulose membranes (Thermo Scientific) by semi-dry blotting. After blocking in TBS buffer (10 mM Tris-HCl pH 7.5, 144 mM NaCl) containing 5% (w/v) bovine serum albumin (BSA), membranes were exposed to the following primary antibodies diluted in TBST (10 mM Tris-HCl pH 7.5, 144 mM NaCl, 0.05% (w/v) Tween20) containing 2% (w/v) BSA: mouse monoclonal α-cmyc 9E10 (1:1000), mouse monoclonal α-HA.11 16B12 (1:1000), rabbit α-BiP (1:25000), rabbit α-aldolase (1:1000), mouse α-mtHSP70 (a gift of Paul E. Englund, Baltimore, MD; diluted 1:1000), and mouse α-nuclear pore complex proteins (MAb414; diluted 1:500). Corresponding secondary antibodies were used at a dilution of 1:20000 (DyLight conjugate, Pierce). Detection of fluorescent antibodies was done using the Odyssey Imaging System (LI-COR).

### C/EPT activity assays and lipid analysis

Activity assays were performed using 80 μg of protein in 100 μl of HKM buffer.

#### CEPT assay

80 μg of protein from sub-cellular fractions were incubated for 50 min at 27 °C in 100 μl assay buffer (50 mM Tris pH 8.0, 10 mM MgCl_2_·6 H_2_O, 0.005% (w/v) Tween20), supplemented with 5 nmoles CDP-choline, 0.9 nmoles [^14^C]CDP-choline, 2.5 nmoles diacylglycerol and 2.5 nmoles alkyl-acylglycerol. After incubation, lipids were extracted twice by addition of 250 μl 1-butanol, centrifugation and isolation of the organic phase.

#### EPT assay

The assay was performed as described for CEPT, with CDP-ethanolamine and [^14^C]CDP-ethanolamine as substrates instead of CDP-choline and [^14^C]-labeled CDP-choline.

#### Thin layer chromatography (TLC)

Lipid classes were separated on silica gel 60 plates (Merck) by one-dimensional TLC using a solvent system composed of chloroform: methanol: acetic acid: water (25:15:4:2, by vol.). Lipid standards were run on each plate alongside the samples to be analysed. Radioactive lipids were detected by scanning the air-dried plate with a radioisotope detector (Berthold Technologies) and quantified using the Rita Star software provided by the manufacturer.

### PE and PC synthesis in TbCEPT RNAi parasites

Metabolic labeling of trypanosomes with [^3^H]choline or [^3^H]ethanolamine was done as described previously^20^. Briefly, 2.5 μCi/ml [^3^H]choline or 0.5 μCi/ml [^3^H]ethanolamine was added to 4 × 10^7^ trypanosomes in culture medium and incubated for 2 h at 27 °C. Cells were harvested, washed once in TBS buffer and lipids were extracted^20^. [^3^H]-labeled PC and PE were analysed by TLC as described above.

## Additional Information

**How to cite this article**: Farine, L. *et al.* Phosphatidylethanolamine and phosphatidylcholine biosynthesis by the Kennedy pathway occurs at different sites in *Trypanosoma brucei*. *Sci. Rep.* **5**, 16787; doi: 10.1038/srep16787 (2015).

## Figures and Tables

**Figure 1:**
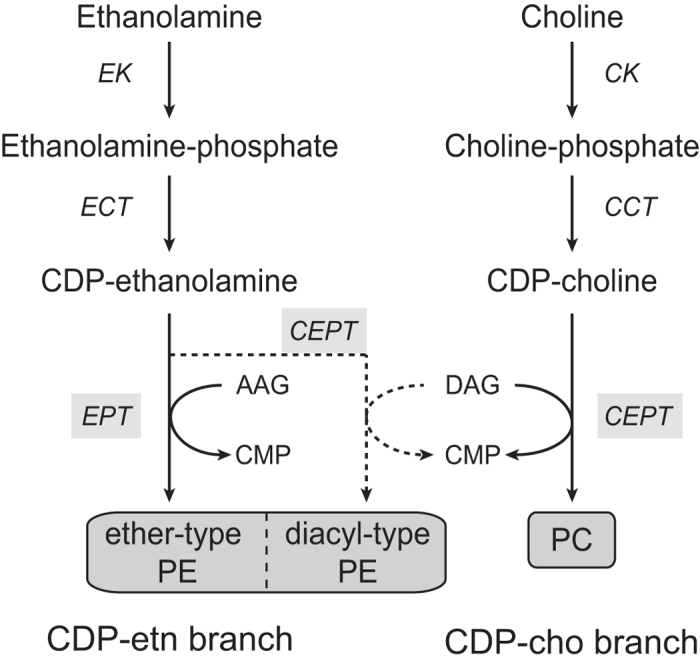
PE and PC synthesis via the Kennedy pathway in *Trypanosoma brucei.* TbEPT synthesizes exclusively ether-type PE using AAG as substrate, while TbCEPT synthesizes both diacyl-type PE and PC using DAG substrate. The *T. brucei* genome lacks a gene encoding CPT. E/CK, ethanolamine/choline kinase; E/CCT, ethanolamine/choline cytidylyltransferase, EPT, ethanolamine phosphotransferase; CEPT, choline/ethanolamine phosphotransferase, AAG, alk-1-enyl-acylglycerol/alkyl-acylglycerol; DAG, diacylglycerol; CMP, cytidine monophosphate.
